# Propionic acid secreted by *Akkermansia muciniphila* alleviates hepatic fibrosis by antioxidant regulation across the gut–liver axis

**DOI:** 10.1093/lifemeta/loaf036

**Published:** 2025-10-08

**Authors:** Lu Zhang, Jing Chen, Si-Jia Ge, Tian-Yi Huang, Xiang Shi, Yu-Yan Chen, Cui-Hua Lu

**Affiliations:** Department of Gastroenterology, Affiliated Hospital of Nantong University, Medical School of Nantong University, Nantong, Jiangsu 226001, China; Department of Gastroenterology, Affiliated Hospital of Nantong University, Medical School of Nantong University, Nantong, Jiangsu 226001, China; Department of Gastroenterology, Affiliated Hospital of Nantong University, Medical School of Nantong University, Nantong, Jiangsu 226001, China; Department of Gastroenterology, Affiliated Hospital of Nantong University, Medical School of Nantong University, Nantong, Jiangsu 226001, China; Department of Gastroenterology, Affiliated Hospital of Nantong University, Medical School of Nantong University, Nantong, Jiangsu 226001, China; Division of Hepatobiliary and Transplantation Surgery, Department of General Surgery, Nanjing Drum Tower Hospital, Affiliated Hospital of Medical School, Nanjing University, Nanjing, Jiangsu 210008, China; Department of Gastroenterology, Affiliated Hospital of Nantong University, Medical School of Nantong University, Nantong, Jiangsu 226001, China

**Keywords:** AKK, gut–liver axis, hepatic fibrosis, TGF-β/SMAD pathway

## Abstract

The gut commensal bacterium *Akkermansia muciniphila* (AKK) has emerged as a candidate for treating liver disorders, yet its therapeutic potential in liver fibrosis remains poorly defined. Here, using a carbon tetrachloride (CCl_4_)-induced murine model, we show that AKK administration markedly attenuates collagen deposition, inflammation, and hepatic injury. AKK restored intestinal barrier integrity, reshaped microbial composition, and enhanced propionic acid transport from the gut to the liver, leading to suppression of hepatic stellate cell activation. Multi-omics profiling revealed that AKK enriched propionate-producing taxa and upregulated key metabolic enzymes, thereby elevating hepatic propionate levels. Supplementation with propionic acid alone recapitulated AKK’s benefits, improving liver function, alleviating extracellular matrix accumulation, and activating the Keap1–Nrf2 antioxidant pathway. Together, our findings identify a microbiota–metabolite axis in which AKK counters liver fibrosis by enhancing propionate-mediated antioxidant regulation, highlighting its therapeutic promise for chronic liver disease.

## Graphical Abstract

**Graphical Abstract:**
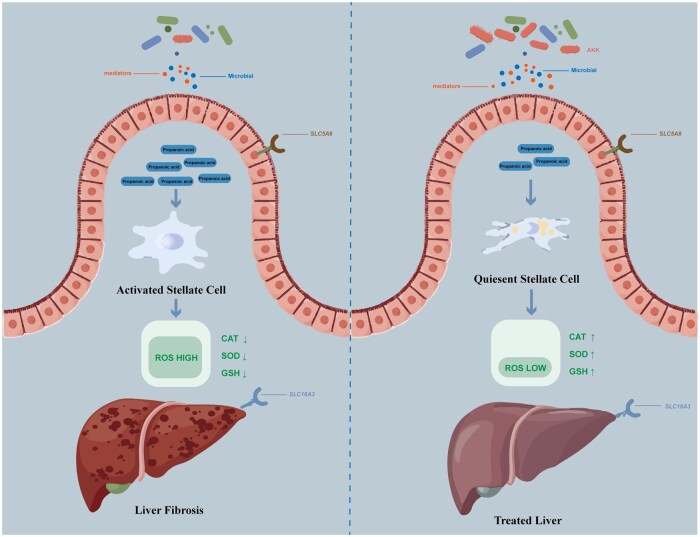

## Introduction

Hepatic fibrosis, serving as the final common pathway of chronic inflammation, is fundamentally driven by the core pathogenic mechanisms of aberrant activation of hepatic stellate cells (HSCs) and uncontrolled extracellular matrix (ECM) deposition. These processes are intricately intertwined with gut microbial homeostasis [1–5]. Recent groundbreaking studies have unveiled that gut microbiota dysbiosis is not merely an accompanying phenomenon of hepatic fibrosis but a pivotal independent factor driving fibrotic progression through gut–liver axis signaling. During fibrogenesis, microbiota imbalance disrupts the intestinal barrier, enabling microbial-derived metabolites (e.g. lipopolysaccharides, secondary bile acids) and viable bacteria to translocate to the liver. These components directly activate HSCs via Toll-like receptors (TLRs) and amplify the expression of pro-fibrotic mediators such as the transforming growth factor-β (TGF-β) and interleukin 17A (IL-17A) [6–8]. Metagenomic analyses in cirrhotic patients further confirm a distinctive “pro-fibrotic microbial signature”, characterized by an enrichment of pro-inflammatory genera (e.g. *Veillonella*, *Streptococcus*) and depletion of protective symbiotic taxa (e.g. *Bacteroides*). This dysbiotic profile demonstrates a positive correlation with hepatic collagen accumulation, thereby positioning gut microbiota modulation as a novel therapeutic target for halting fibrotic progression [7, 9].

*Akkermansia muciniphila* (*AKK*) is one of the most abundant resi­dent probiotic bacteria in human gut, exhibiting activity against various immune dysfunctions and metabolic disorders. For instance, *AKK* can induce gut adaptive immune responses during homeostasis and produce bioactive molecules capable of modulating distal cellular activities, intervening in pathological dev­elopments [10]. The discovery of a novel bioactive tripeptide Arg-Lys-His (RKH), produced by live *AKK*, has expanded its pathophysiological role, potentially serving as a promising therapeutic approach for lethal sepsis [11]. However, whether and how *AKK* exerts its anti-fibrotic effects in the liver remains unexplored.

This investigation was designed to systematically evaluate the anti-fibrotic efficacy of *AKK* administration in a carbon tetrachloride (CCl_4_)-induced murine hepatic fibrosis model, a well-established paradigm that recapitulates critical features of human fibrogenesis through sustained HSC activation. Our experimental framework was structured along two investigative axes: primary evaluation of macroscopic therapeutic outcomes and secondary mechanistic exploration through multi-omics profiling.

## Results

### Gastric infusion of *AKK* mitigates liver injury and collagen deposition in mice with liver fibrosis

In this item, we explored the composition of the gut microbiota in fecal samples from mice affected by hepatic fibrosis. Analysis revealed two distinct clusters corresponding to CCl_4_-treated and *AKK*-infused groups, highlighting significant variances in microbial community structures between them (Supplementary Fig. S1a). Further, analysis of alpha-diversity, as measured by operational taxonomic units (OTUs) and the Shannon index, revealed that gastric administration of *AKK* enhanced the richness and diversity of the gut microbiota in fibrotic mice (Supplementary Fig. S1b and c). This improvement in microbial community structure is depicted in Supplementary Fig. S1d. Notably, linear discriminant analysis effect size (LEfSe) analysis illustrated alterations in the microbial composition of mice with liver fibrosis after gastric infusion ­(Supplementary Fig. S1e).

**Figure 1:**
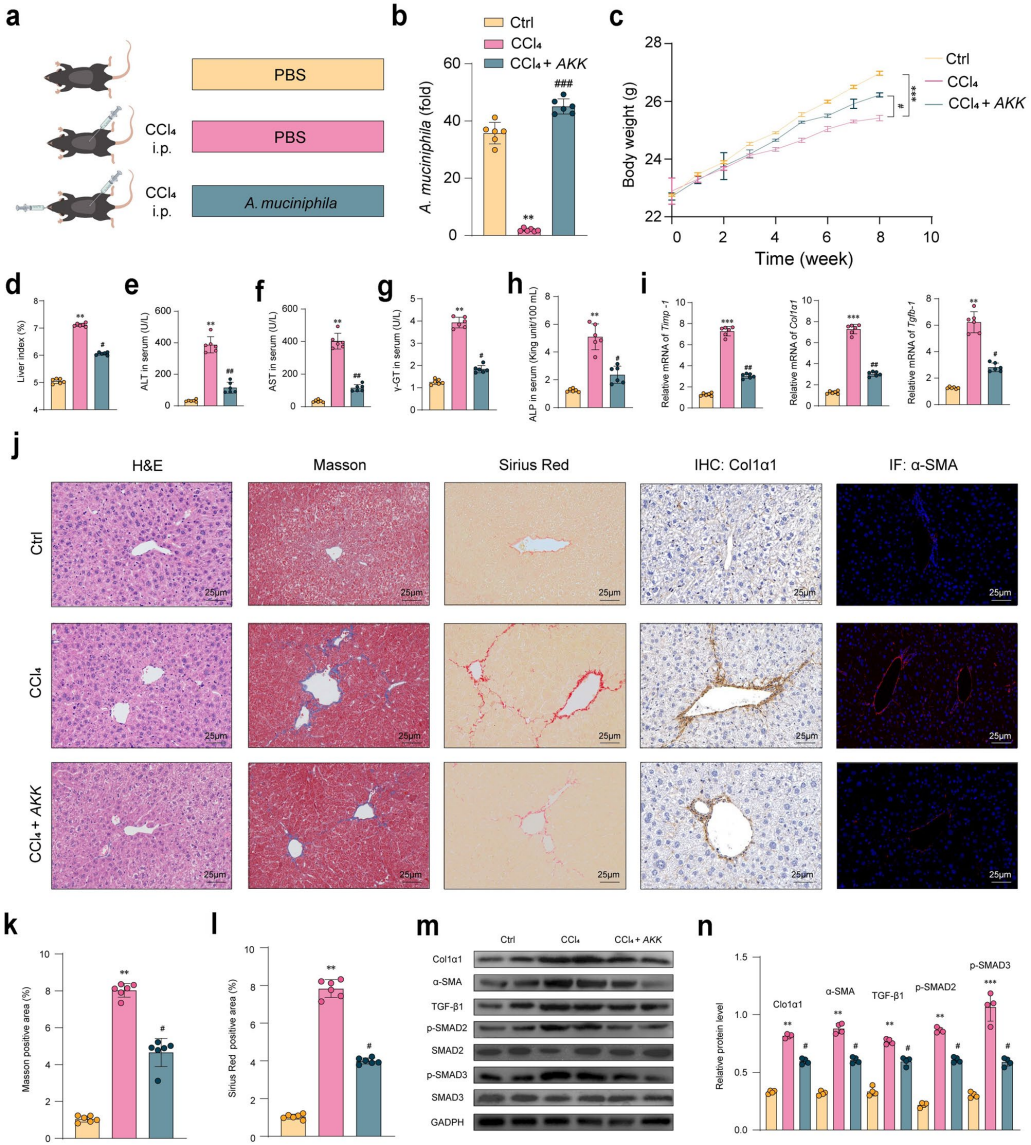
AKK mitigates liver injury and collagen deposition in mice with liver fibrosis. (a) Diagram of the experimental scheme. (b) Abundance of *AKK* in feces. (c) Body weight of animals recorded weekly during the experimental period. (d) Liver index (liver weight/body weight). (e–h) Serum ALT (e), AST (f), γ-GT (g), and ALP (h) content (*n *= 6). (i) Relative mRNA levels of *Timp-1*, *Clo1α1*, and *Tgfb1* (*n *= 6). (j) Representative images of H&E, Masson, Sirius Red, IHC, and immunofluorescence (IF) staining (Scale bar = 25 μm) in the liver (*n *= 6). (k and l) Liver collagen deposition index (*n *= 6). (m and n) Fibrosis-related markers such as TIMP-1, α-SMA, and Col1α1 and TGF-β1 protein expression assayed by western blotting. Data are presented as the mean ± SEM. Statistical significance was determined using ANOVA for multiple-group comparisons. *P < 0.05, ^**^P < 0.01, ^***^P < 0.001, compared with the control group. ^#^*P *< 0.05, ^##^*P *< 0.01, compared with the model group.

We then assessed the therapeutic efficacy of *AKK* in treating liver fibrosis using a CCl_4_-induced mouse model (Fig. 1a). In comparison to the CCl_4_ group mice, quantitative real-time polymerase chain reaction (qRT-PCR) analysis demonstrated a substantial 27-fold increase in *AKK* abundance within the intestines of *AKK*-treated mice (Fig. 1b). The experimental results demonstrated that oral administration of *AKK* significantly alleviated weight loss and liver enlargement induced by CCl_4_ in mice (Fig. 1c and d). Administration of *AKK* significantly ameliorated liver damage in mice treated with CCl_4_, evidenced by reduced plasma levels of liver-specific enzymes (aspartate aminotransferase [AST], alanine aminotransferase [ALT], γ-glutamyl transpeptidase [γ-GT], and alkaline phosphatase [ALP]) (Fig. 1e−h). This treatment also notably decreased the expression of key genes associated with steatosis and inflammation, such as tissue inhibitor of metalloproteinases-1 (*Timp-1*), collagen1α1 (*Col1α1*), and *Tgfb-1* (Fig. 1i). Further, histopathological assessments confirmed the therapeutic impact of *AKK* on fibrosis phenotypes in mice, including hepatocyte damage, inflammatory responses (hematoxylin and eosin [H&E] staining), ECM accumulation (Masson and Sirius Red staining), and markers of fibrosis (immunohistochemistry [IHC] for Col1α1 and for α-smooth muscle actin [α-SMA]) (Fig. 1j–l). Using western blot analysis, we found that *AKK* supplementation led to reduced protein expression levels of liver fibrosis markers, specifically α-SMA and Col1α1, compared to the group induced with CCl_4_ alone (Fig. 1m). *AKK* supplementation following CCl_4_ induction effectively inhibited the activation of the TGF-β/small mother against decapentaplegic 2/3 (SMAD2/3) pathway in hepatic fibrosis (Fig. 1m and n). In this study, we provided evidence for the protective effects of *AKK* against CCl_4_-induced liver fibrosis. Furthermore, the advantageous impacts of *AKK* are linked to its ability to modulate the gut microbiota composition.

### *AKK* markedly improves intestinal barrier function in CCl_4_-exposed mice

**Figure 2:**
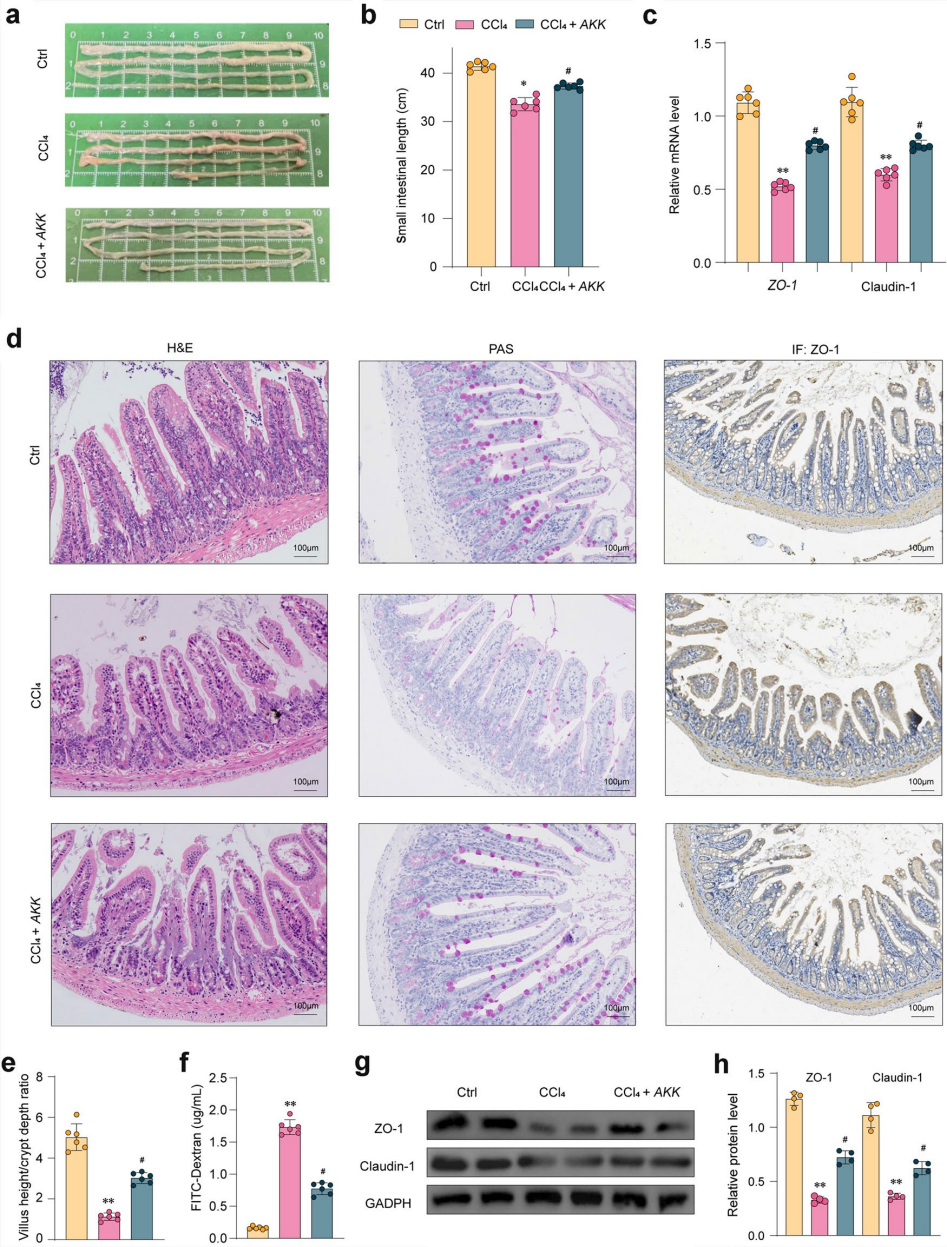
Effects of *AKK* supplementation on the intestinal barrier impairment in the ileum of CCl_4_-exposed mice. (a and b) Representative photos of small intestines captured by camera (a) and the length of small intestine measured (b). (c) Relative mRNA expression of *ZO-1* and Claudin-1 (*n *= 6). (d) Representative images of H&E, PAS, and IHC staining (Scale bar = 100 μm) in the ileum (*n *= 6). (e) Statistical analysis of the villus height and crypt depth (*n *= 6). (f) Assessment of FITC-dextran permeability in mouse (*n *= 6). (g and h) The levels of ZO-1 and Claudin-1 quantified by western blot analysis. Data are presented as the mean ± SEM. Statistical significance was determined using ANOVA for multiple-group comparisons. ^*^P < 0.05, ^**^P < 0.01, compared with the control group. ^#^*P *< 0.05, compared with the model group.

This protective effect of *AKK* on the intestinal barrier is crucial for maintaining gut health and has important implications for liver disease prevention and treatment. *AKK* pretreatment significantly increased the length of the small intestine (Fig. 2a and b). Moreover, the results demonstrated a significant decrease in the mRNA expression of zonula occludens-1 (*ZO-1*) and claudin-1 after CCl_4_ treatment (*P *< 0.05) (Fig. 2c). In Fig. 2d, after CCl_4_ treatment, the mucosal layer exhibited extensive edema, loose arrangement of connective tissue, and abundant infiltration of inflammatory cells. Following *AKK* treatment, the mucosal epithelium remained intact, with normal cell morphology and no significant inflammatory changes. Immunohistochemical analysis of intestinal tight junction markers revealed higher levels of ZO-1 in the *AKK* intervention group compared to the CCl_4_ group. Figure 2e demonstrates a significant reduction in the villus height to crypt depth ratio following CCl_4_ administration (*P *< 0.01). Following *AKK* treatment, this ratio was significantly reversed. Furthermore, we assessed intestinal mucosal permeability by administering fluorescein isothiocyanate (FITC)-dextran via gavage in mice. The results showed that compared to the control group, the CCl_4_ group exhibited significant barrier disruption and a substantial increase in FITC-dextran entering circulation. However, administration of *AKK* resulted in reduced mucosal permeability and alleviation of barrier damage (Fig. 2f). Immunohistochemical analysis through western blot of intestinal tight junction markers showed higher levels of ZO-1 and claudin-1 in the *AKK* intervention group compared to the CCl_4_ group (Fig. 2g and h). Therefore, we hypothesize that *AKK* pretreatment exerts beneficial effects on CCl_4_-induced intestinal barrier dysfunction and liver fibrosis by modulating the gut microbiota composition and its metabolites.

### *AKK* increases liver propionic acid levels by promoting propionic acid transport from the intestine

**Figure 3:**
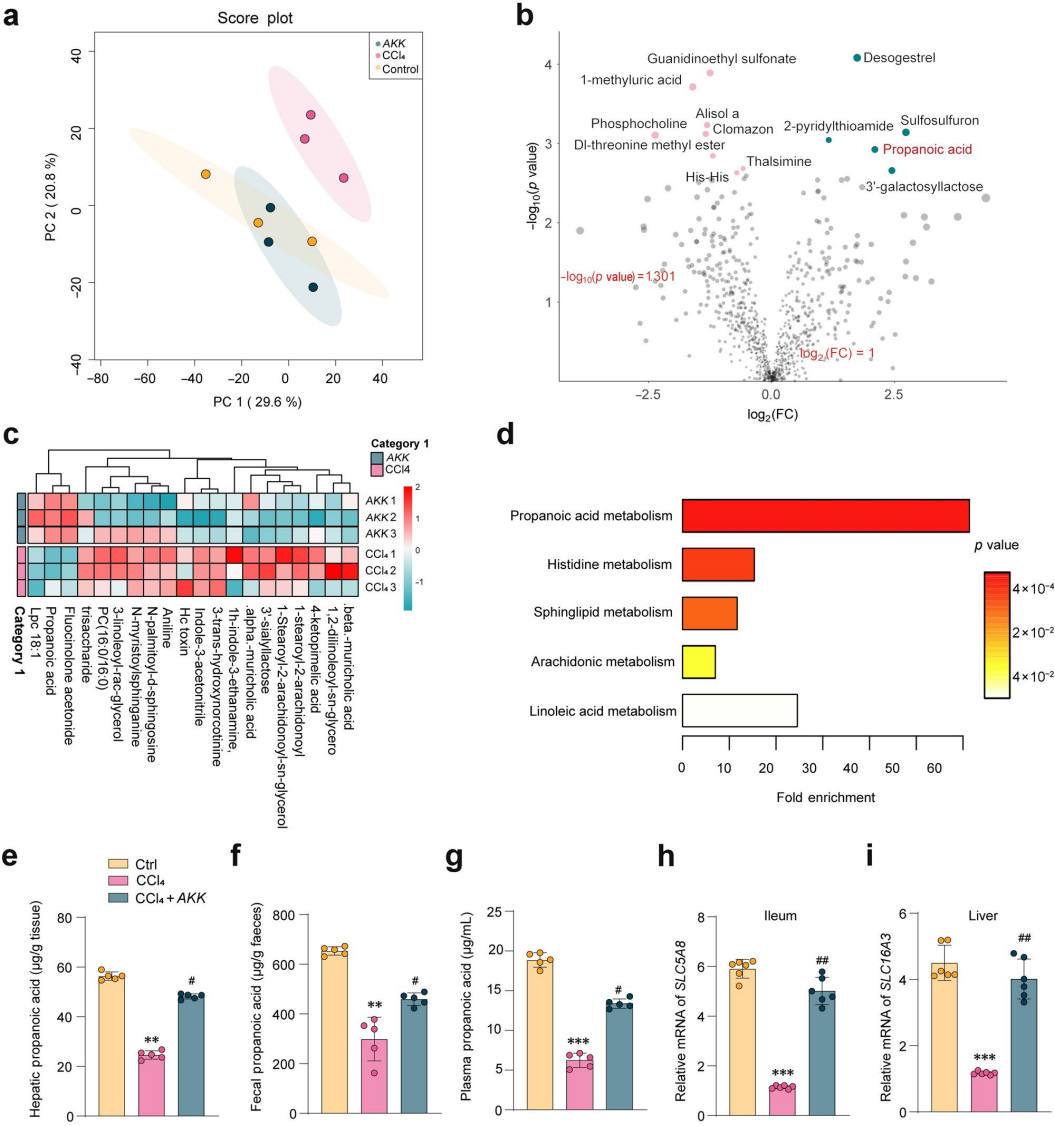
AKK increases propionic acid levels in the livers of hepatic fibrosis mice. (a) PCA. (b and c) Identification of significant differential metabolites in CCl_4_ or *AKK*-treated hepatic fibrosis mice by LC-MS analysis. (d) Annotation of identified significant metabolites in the CCl_4_ or *AKK*-treated groups by metabolomics trend analysis. (e–g) Propionic acid levels in the livers (e), feces (f), and plasma (g) of mice (*n *= 5). (h) Gene expression of *Slc52a1* in the ileum of mice (*n *= 5). (i) Gene expression of *Slc52a2* in the livers of mice (*n *= 5). Data are presented as the mean ± SEM. Statistical significance was determined using ANOVA for multiple-group comparisons. ^**^P < 0.01, ^***^P < 0.05, compared with the control group. ^#^*P *< 0.05, ^##^*P *< 0.01, compared with the model group.

To elucidate the potential mechanism underlying the anti-liver fibrosis activity of *AKK*, our metabolomic analysis aimed to profile the liver metabolome, with principal component analysis (PCA) highlighting marked compositional distinctions between the CCl_4_-treated, control, and *AKK*-treated groups (Fig. 3a). Metabolites driving these differences (as depicted in Supplementary Fig. S2), were identified and linked to crucial liver functions such as short-chain fatty acid (SCFA) metabolism, lipid degradation, and bile acid synthesis. Notably, compared to the CCl_4_ group, *AKK*-treated mice demonstrated a significantly altered metabolome, characterized by elevated levels of these metabolites (Fig. 3b). Furthermore, the liver metabolome of the *AKK*-treated group showed more significant differences compared to mice with liver fibrosis induced by CCl_4_ alone, with a total of 20 significantly different metabolites detected, including propionic acid (Fig. 3c). Upon further examination of these cohorts, noteworthy metabolites emerged, including propionic acid, histidine, sphingolipid, arachidonic acid, and linoleic acid. These metabolites were classified based on the Kyoto Encyclopedia of Genes and Genomes (KEGG) database (Fig. 3d). Quantitative assessment of liver propionic acid levels demonstrated a significant increase following *AKK* treatment, suggesting a potential association between *AKK*-mediated improvement of liver fibrosis and propionic acid, as a candidate therapeutic for liver fibrosis.

Furthermore, we measured propionic acid levels in the liver, plasma, and feces. *AKK* treatment effectively increased propionic acid levels in all three of these compartments (Fig. 3e–g). The gene expression levels of propionic acid transporters (solute carrier gene family 5a, member 8 [*SLC5A8*] or solute carrier family 16, member 3 [*SLC16A3*]) in the ileum or liver elevated levels in *AKK*-treated mice (Fig. 3h and i).

### Supplementation with *AKK* enhances propionic acid-metabolizing microbiota and the expression of related enzymes

**Figure 4:**
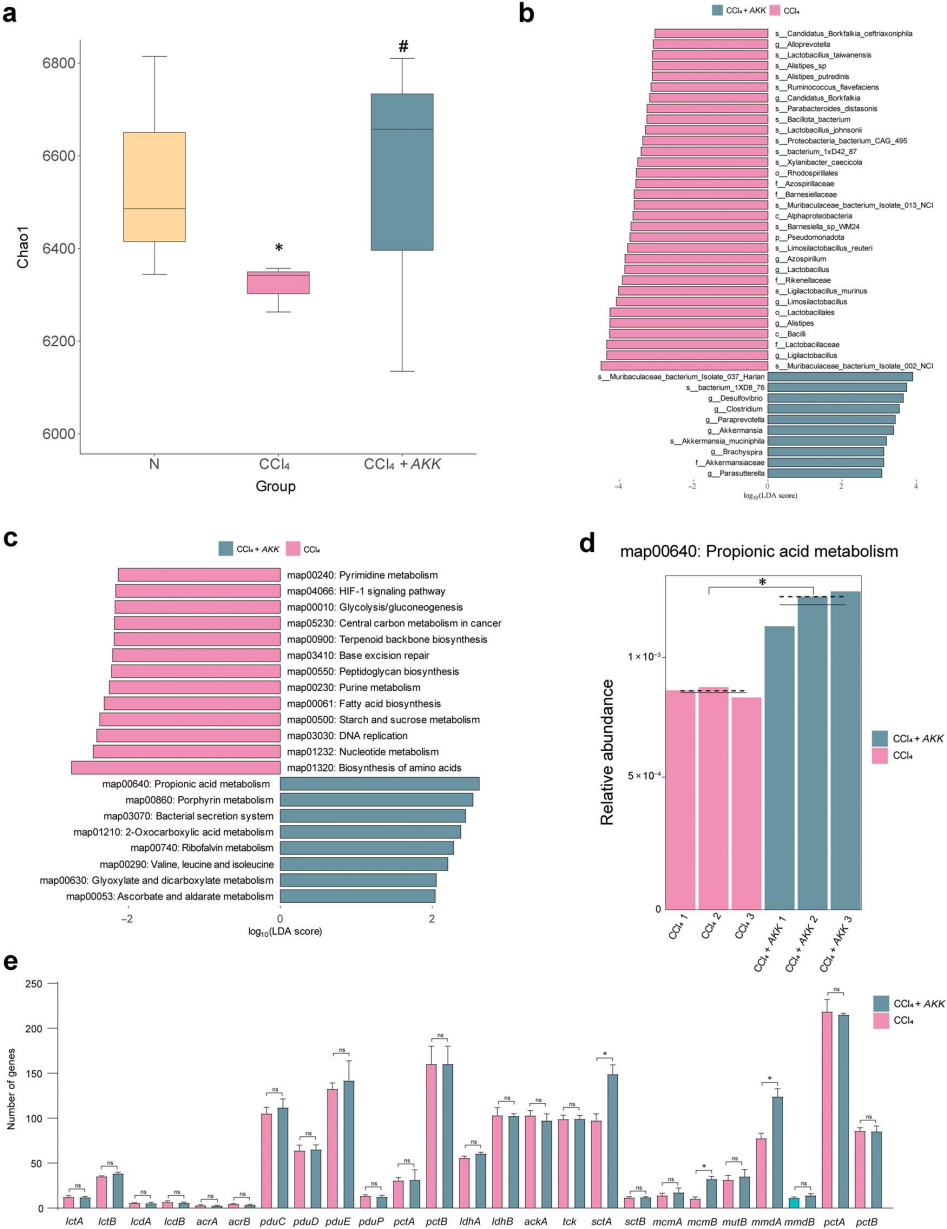
AKK supplementation enriches propionic acid-metabolizing bacteria and enhances the expression of related enzymes. (a) Chao1 index. (b) LDA score based on LEfSe analysis in phylum level (LDA > 2) (*n *= 3). (c) LEfSe analysis in KEGG pathway (LDA > 2) (*n *= 3). (d) Relative abundance. (e) Number of genes in propionic acid metabolism. Data are presented as the mean ± SEM. Statistical significance was determined using ANOVA for multiple-group comparisons. ^**^P < 0.01, ^*^P < 0.05, compared with the CCl_4_ group.

To elucidate the potential mechanism by which *AKK* combats liver fibrosis through the enhancement of propionic acid metabolism, we conducted further metagenomic analysis to characterize the genomic features of the propionic acid-metabolizing microbiota under hepatic fibrosis conditions with *AKK* supplementation. PCA revealed significant compositional differences between the CCl_4_-treated, control, and *AKK*-treated groups (Supplementary Fig. S3). Initially, our metagenomic analysis showed a marked increase in Chao1 index following *AKK* supplementation, indicating a notable improvement in both microbial abundance and gene expression (Fig. 4a). Additionally, results from the LEfSe algorithm indicated that *AKK* supplementation facilitated gut colonization, with a higher relative abundance compared to the CCl_4_-treated group (Fig. 4b). Further functional annotation of the metagenomic data revealed an increased expression of pathways associated with propionic acid metabolism after *AKK* supplementation (Fig. 4c and d). Given the involvement of various hydrolases in propionic acid metabolism, further analysis of gene expression in microbial pathways related to propionic acid showed increased abundance of key genes encoding propionic acid hydrolases, including *sctA*, *mcmB*, and *mmdA* (Fig. 4e). These findings provide insight into how *AKK* supplementation may treat liver fibrosis by improving the composition and gene expression of propionic acid-metabolizing microbial taxa.

### *AKK* alleviates liver fibrosis in mice by improving propionic acid metabolism

**Figure 5:**
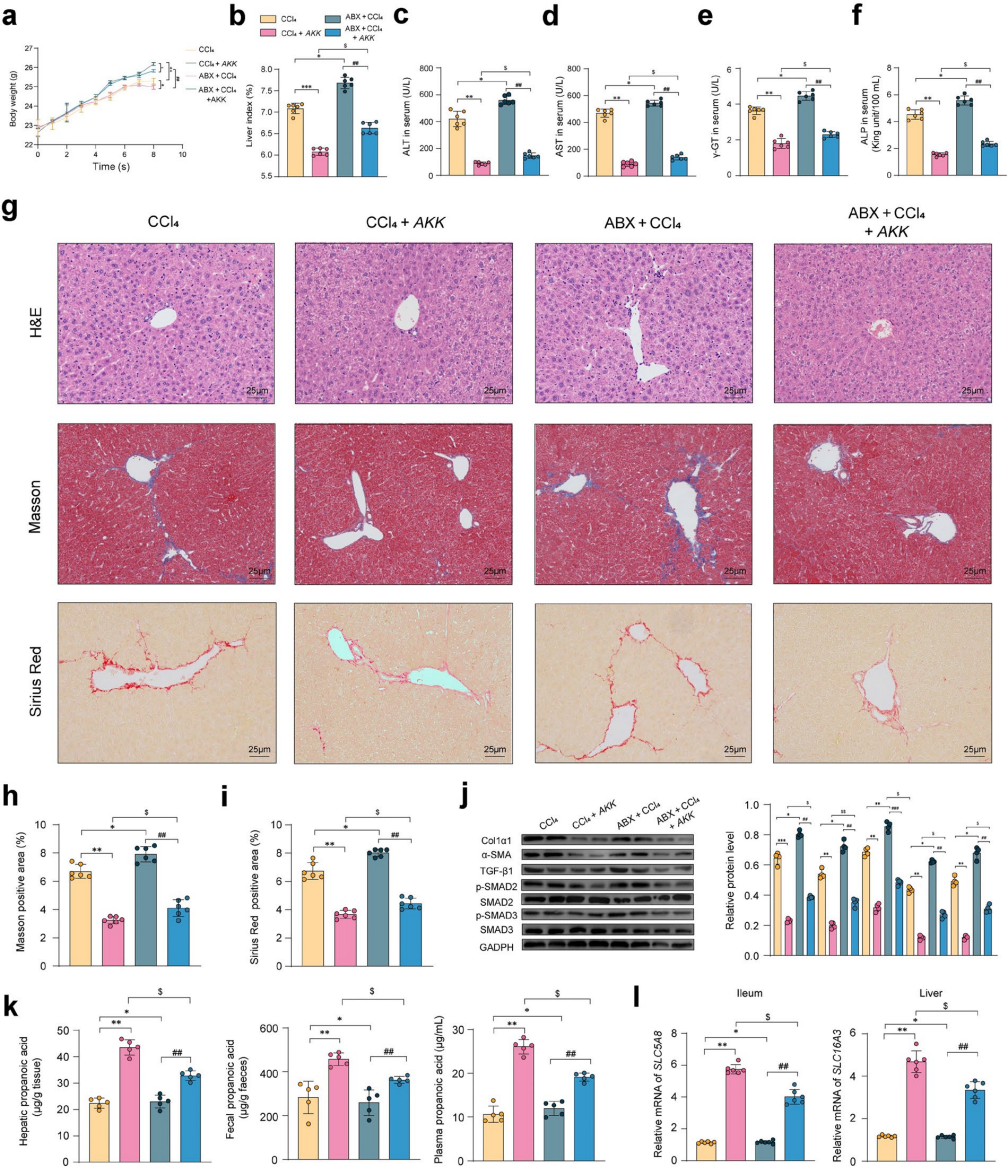
AKK alleviates liver fibrosis in mice by improving propionic acid metabolism. (a) Body weight of animals recorded weekly during the experimental period. (b) Liver index (*n *= 6). (c–f) Serum content of ALT (c), AST (d), γ-GT (e), and ALP (f) (*n *= 6). (g) Representative images of H&E, Masson, and Sirius Red staining (scale bar = 25 μm) in the liver (*n *= 6). (h and i) Liver collagen deposition index (*n *= 6). (j) Fibrosis-related markers such as TIMP-1, α-SMA, and Col1α1 and TGF-β1 protein expression assayed by western blotting. (k) Propionic acid levels in the livers, feces, and plasma of mice (*n *= 6). (l) Gene expression of *Slc52a1* and *Slc52a2* in mice (*n *= 6). Data are presented as the mean ± SEM. Statistical significance was determined using ANOVA for multiple-group comparisons. CCl_4_-induced hepatic fibrosis (CCl_4_), ABX + CCl_4_, or a combination of ABX + CCl_4_ plus *AKK* (ABX + CCl_4_ + *AKK*) were used to assess liver metabolomics and the indicated assays. ^**^  *P *< 0.01, ^***^  *P *< 0.05, compared with the CCl_4_ group. ^#^  *P *< 0.05, ^##^  *P *< 0.01, compared with the ABX + CCl_4_ group. ^$^  *P *< 0.05, compared with the CCl_4_ + *AKK* group.

Through measurements of mouse body weight and liver weight, we observed that antibiotic (ABX) attenuated the effects of *AKK* on CCl_4_-induced weight loss and liver enlargement (Fig. 5a and b). We determined the levels of liver-specific enzymes in plasma, which indicated that ABX treatment partially limited the liver function improvement effects of *AKK* in the context of liver fibrosis (Fig. 5c–f). Results from H&E, Masson, and Sirius Red staining showed that *AKK* and ABX combination treatment exhibited a more significant alleviation of liver fibrosis-related collagen deposition compared to CCl_4_-induced mice treated with ABX alone (Fig. 5g–i). Additionally, in the combination treatment group, ABX was found to promote the activation of the TGF-β/SMAD2/3 pathway in liver fibrosis, suggesting that ABX may influence the impact of *AKK* metabolites on the TGF-β/SMAD2/3 pathway in hepatic fibrosis (Fig. 5j).

We conducted an assessment of propionic acid concentration in the plasma and feces. The findings revealed that *AKK* intervention notably elevated these levels in both media. Conversely, ABX intervention amplified the propionic acid concentration in the feces while diminishing it in the plasma and liver (Fig. 5k). *AKK* heightened the expression levels of *SLC5A8* and *SLC16A3*, while ABX treatment restrained propionic acid transporter, partially counteracting the stimulatory effect of *AKK* on propionic acid transporter gene expression (Fig. 5l).

### Propionic acid supplementation alleviates oxidative stress in mice with hepatic fibrosis

**Figure 6:**
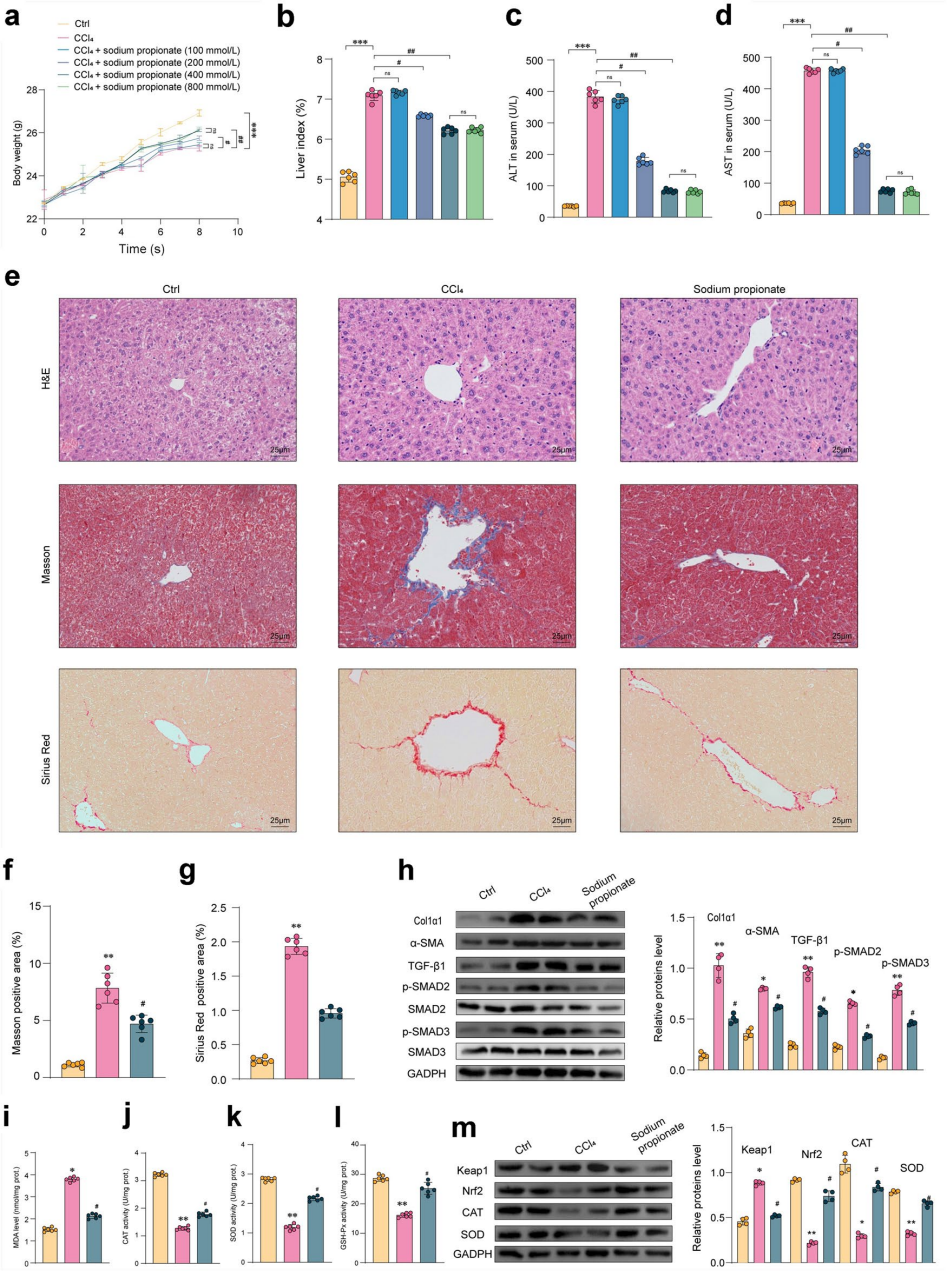
Propionic acid alleviates liver fibrosis in mice and reduces oxidative stress. (a) Body weight of animals recorded weekly during the experimental period. (b) Liver index (*n *= 6). (c and d) Serum content of ALT (c) and AST (d) (*n *= 6). (e) Representative images of H&E, Masson, and Sirius Red staining (Scale bar = 25 μm) in the liver. (f and g) Liver collagen deposition index (*n *= 6). (h) Fibrosis-related markers such as TIMP-1, α-SMA, and Col1α1 and TGF-β1 protein expression assayed by western blotting. (i–l) MDA level (i), CAT activity (j), SOD activity (k), and GSH-Px activity (l) (*n *= 6). (m) Oxidative stress-related Keap1/Nrf2 pathway protein expression assayed by western blotting. Data are presented as the mean ± SEM. Statistical significance was determined using ANOVA for multiple-group comparisons. ^**^P < 0.01, ^***^P < 0.05, compared with the control group. ^#^*P *< 0.05, compared with the model group.

In this study, we first determined the therapeutic concentration of propionic acid by exploring a concentration range of 100, 200, 400, and 800 mmol/L. We found that 400 mmol/L significantly alleviated CCl_4_-induced weight loss and liver enlargement in mice, compared to 200 mmol/L. However, there was no significant difference between 800 mmol/L and 400 mmol/L in terms of alleviating weight loss and liver enlargement induced by CCl_4_ (Fig. 6a and d). Additionally, propionic acid demonstrated a therapeutic effect on liver function markers (ALT and AST) in mice, indicating a relief of fibrosis, which was also validated (Fig. 6c and d). Therefore, we selected 400 mmol/L as the therapeutic concentration for propionic acid, and subsequent histopathological analysis further confirmed its beneficial effects on other phenotypes of fibrosis in mice (Fig. 6e–g). Subsequently, western blot analysis revealed that supplementation of propionic acid resulted in decreased expression levels of liver fibrotic markers such as α-SMA and Col1α1 compared to the CCl_4_-induced model group (Fig. 6h). Furthermore, supplementation of propionic acid after CCl_4_ induction impeded the activation of the TGF-β/SMAD2/3 pathway in liver fibrosis (Fig. 6h).

Propionic acid supplementation exhibited an increase in the antioxidant activity within these cells, suggesting its possible role in counteracting oxidative stress under *in vivo* circumstances. Key antioxidant markers, such as catalase (CAT), superoxide dismutase (SOD), and glutathione peroxidase (GSH-Px), displayed a significant rise in liver fibrosis mice after propionic acid supplementation (Fig. 6i–l). In mice with CCl_4_-infused hepatic fibrosis, liver tissue showed a significant reduction in the expression levels of CAT, SOD2, and nuclear factor erythroid-2-related factor 2 (Nrf2) compared to the control group. Contrastingly, propionic acid supplementation resulted in a distinct upregulation of these proteins (Fig. 6m). By activating the Kelch-like ECH associated protein 1 (Keap1)/Nrf2 pathway and reversing CCl_4_-induced oxidative stress, propionic acid actively mitigates liver fibrosis. Therefore, propionic acid supplementation may have potential therapeutic efficacy in managing hepatic fibrosis by targeting oxidative stress reduction and redox balance restoration.

### Propionic acid directly inhibits the activation of HSCs and oxidative stress

**Figure 7:**
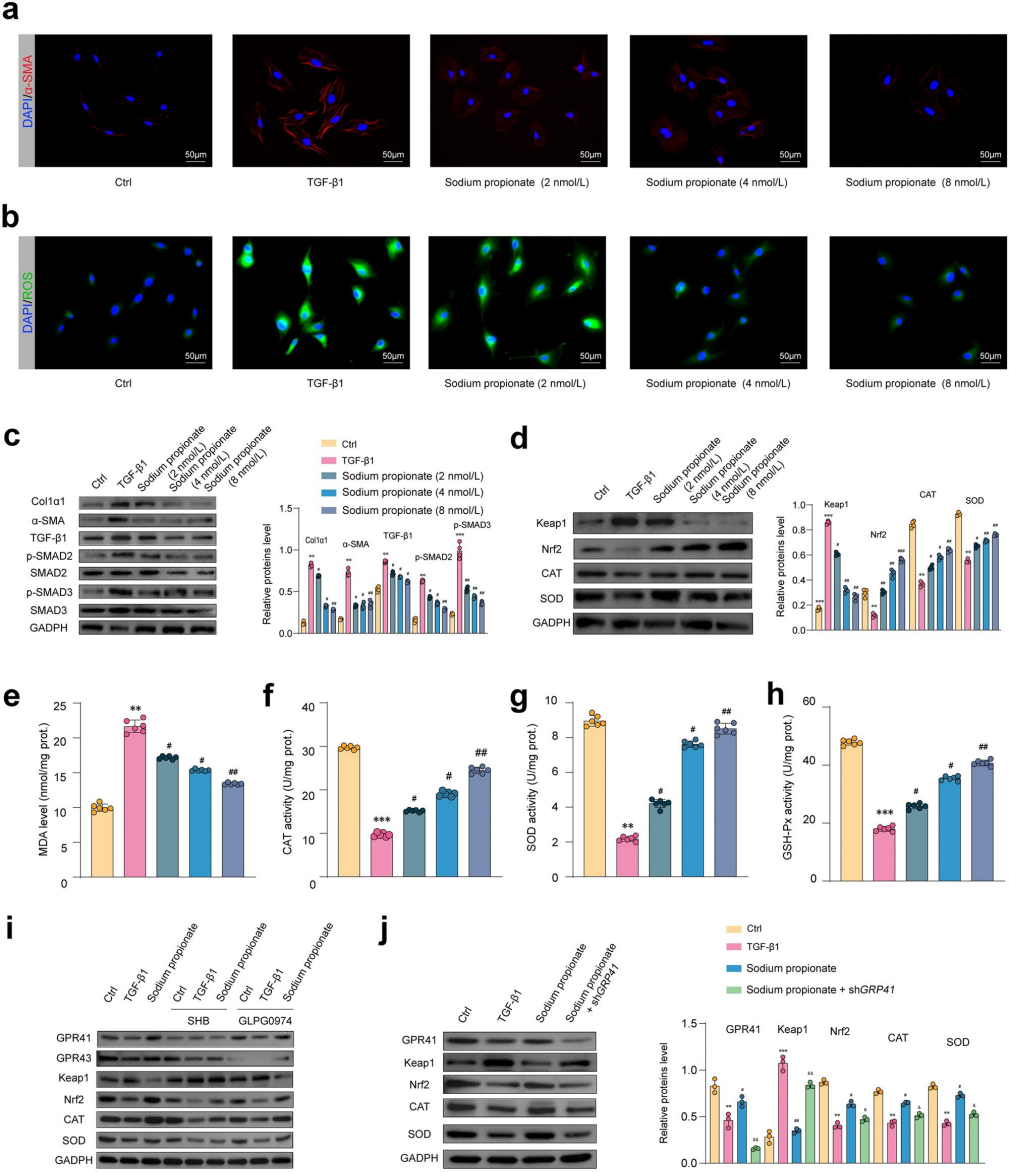
Propionic acid inhibits the activation of HSCs and oxidative stress. (a) Immunofluorescence analysis (Scale bar = 50 μm). (b) ROS visualized by microscopy using the 2’,7’-dichlorodihydrofluorescein diacetate (DCFH-DA) probe at a final concentration of 10 μmol/L (Scale bar = 50 μm). (c) Fibrosis-related markers such as TIMP-1, α-SMA, and Col1α1 and TGF-β1 protein expression assayed by western blotting. (d) Oxidative stress-related Keap1/Nrf2 pathway protein levels assayed by western blotting. (e–h) MDA level (e), CAT activity (f), SOD activity (g), and GSH-Px activity (h). (i) *In vitro* experiments conducted to assess the effects of TGF-β, GPR41 inhibitor (SHB), GPR43 inhibitor (GLPG0974), and propionic acid pretreatment on oxidative stress-related Keap1/Nrf2 signaling pathway in LX2 cells through immunoblot analysis. (j) Western blot analysis of oxidative stress-related Keap1/Nrf2 signaling pathway performed in LX2 cells transfected with sh*GPR41*, followed by stimulation with TGF-β and pretreatment with propionic acid. Data are presented as the mean ± SEM. Statistical significance was determined using ANOVA for multiple-group comparisons. ^**^P < 0.01, ^***^P < 0.05, compared with the control group. ^#^*P *< 0.05, ^##^*P *< 0.01, compared with the model group.

In our study, we exposed LX2 cells to propionic acid under TGF-β1 induction. Through western blot analysis, we observed a decrease in the expression levels of hepatic fibrosis markers such as α-SMA and Col1α1 when supplementing propionic acid compared to TGF-β1 group (Fig. 7a). Furthermore, propionic acid supplementation hindered the activation of the TGF-β/SMAD2/3 pathway in hepatic fibrosis (Fig. 7c).

Propionic acid demonstrated inhibitory effects on oxidative stress, as evidenced by the reduction in the levels of reactive oxygen species (ROS) (Fig. 7b). Moreover, supplementation of propionic acid led to a significant increase in representative antioxidant biomarkers in activated LX2 cells, such as CAT, SOD, and GSH-Px levels (Fig. 7d–g). Propionic acid supplementation markedly upregulated the protein expression of CAT, SOD2, and Nrf2, with a dose-dependent trend (Fig. 7h). It is established that propionic acid exerts its effects primarily through G protein-coupled receptor-mediated signaling, with GPR41 and GPR43 receptors playing critical roles. To elucidate the mechanisms underlying the suppression of HSC activation by propionic acid, LX2 cells were treated with either a GPR41 inhibitor (SHB), a GPR43 inhibitor (GLPG0974), or three short-hairpin RNA (shRNA) lentiviruses targeting *GPR41* (Supplementary Fig. S4a and b). Western blot analysis revealed that propionic acid inhibited the activation of the Keap1/Nrf2 signaling pathway in a GPR41-dependent manner (Fig. 7i and j; Supplementary Fig. S4a). Complementary *in vitro* and *in vivo* experiments further confirmed the role of propionic acid in suppressing the activation of HSCs.

## Discussion

The gut microbiota and its byproducts significantly influence host digestion, metabolism, and immunity—factors crucial to human health and disease evolution [12]. Imbalances in the gut microbiota have a close relationship with the hepatic milieu, frequently causing chronic inflammation and fibrosis in the liver [13]. The disruption of the intestinal barrier, dietary components, gut microbiota, and their metabolites, including bile acids, contributes to complex host–microbiota interactions that can either maintain health or promote disease [14]. Additionally, it is evident that compromised intestinal barrier function worsens advanced liver disease, specifi­cally cirrhosis, by facilitating the translocation of numerous bacteria or pathogens and their byproducts to the diseased liver [15]. The dysbiosis-induced hepatic environment often serves as a trigger for chronic inflammation and fibrosis. Hence, targeting hepatic fibrosis therapy based on the gut–liver axis theory holds crucial therapeutic potential, and investigating the mechanisms of action of the gut microbiota and its metabolites in hepatic fibrosis progression is an important area of research.

Our experimental findings demonstrate that *AKK* supplementation significantly alleviated hepatic inflammation, mitigated liver injury, and reduced ECM deposition in murine models of liver fibrosis, thereby meeting recommended criteria for anti-fibrotic interventions. Notably, the therapeutic efficacy of *AKK* in attenuating ECM accumulation and fibrotic progression persisted following ABX perturbation of gut microbiota. Mechanistic investigations revealed that *AKK* administration enhanced enterohepatic translocation of propionic acid, resulting in suppression of TGF-β/SMAD signaling cascade and concomitant attenuation of oxidative stress, ultimately ameliorating hepatic fibrogenesis. This study elucidates the previously unrecognized role of propionic acid in counteracting HSC activation and establishes novel evidence for its regulatory function in redox homeostasis during fibrotic progression.

Studies involving rodents and humans have linked metabolic disorders such as obesity, type 2 diabetes, metabolic dysfunction-associated fatty liver disease, and cardiovascular diseases to a reduced presence of *AKK* [16]. Numerous investigations have been undertaken to shed light on the metabolic mechanisms through which *AKK* modulates energy expenditure and signal transduction. Researchers have pinpointed that a membrane protein purified from *AKK* can enhance intestinal barrier functionality and recapitulate some benefits of the bacterium [17]. *AKK* also plays a significant role in treating chronic liver injury. It was found that oral administration of live and heat-inactivated mucoid streptococci, along with their extracellular vesicles, normalized the composition of fecal-targeted bacteria, improved intestinal permeability, regulated inflammatory responses, and subsequently prevented liver injury in mice subjected to high-fat diet (HFD)/CCl_4_ administration [18]. *AKK* intake by patients with alcoholic liver disease reduces liver damage by improving mucus thickness and tight junction expression to prevent intestinal leakage [19]. However, the specific mechanisms underlying the therapeutic effects of intestinal *AKK* on target tissues remain unclear. This study found that the levels of metabolites such as propionate in the livers of mice treated with *AKK* were altered. Quantitative analysis revealed that *AKK* significantly increased the levels of propionate in the feces, plasma, and livers of mice, and further investigation of propionate transporters confirmed the role of *AKK* in promoting propionate transport. Notably, ABX treatment reduced the levels of propionate in the feces, plasma, and liver, while the combined treatment of ABXs and *AKK*, though attenuating this effect, still resulted in plasma propionate levels higher than those in the *AKK*-only treatment group. This suggests that gut microbiota, in addition to *AKK*, plays a crucial role in the synthesis and transport of propionate. Beyond propionate, *AKK* treatment also led to elevated levels of other metabolites in the liver, which may collectively contribute to the antifibrotic effects of *AKK*, either independently or synergistically. At the very least, this study provides a plausible explanation for the antifibrotic properties of *AKK* through its mediation of increased hepatic propionate levels. These data offer new insights into the underlying mechanisms of *AKK*’s *in vivo* therapeutic effects.

SCFAs are metabolic products of the gut microbiota, serving as a major energy source for intestinal epithelial cells [20]. Additionally, they act as important signaling molecules, regulating various cellular processes such as inflammation and homeostasis of cell proliferation via both receptor-dependent and independent pathways [21]. The pre-intake of *Lactobacillus plantarum* J26 can elevate SCFA levels in the gut, which, through the gut–liver axis, reach the liver, and modulate the Nrf2 signaling pathway to attenuate oxidative stress damage, thereby maintaining mitochondrial homeostasis and autophagic balance, while reducing apoptosis [22]. Propionic acid, a key component of SCFAs, exerts anti-inflammatory effects and can also promote the release of host antimicrobial peptides, contributing to its antibacterial activity [23]. Previous studies have shown that propionic acid regulates mitochondrial fission and the dynamic balance of autophagy, thereby maintaining mitochondrial homeostasis in neurons and mitigating the pathological progression of Alzheimer’s disease [24]. Oxidative stress is a crucial factor in liver damage, particularly in the progression of liver fibrosis. For example, supplementation with vitamin B2 significantly reduces malondialdehyde (MDA) levels in the livers of mice induced by CCl_4_, while repairing the antioxidant defense system [25]. This study reveals that propionate exerts anti-fibrotic effects in the liver by modulating redox balance and inhibiting lipid peroxidation. Propionate enhances the activity of liver antioxidant enzymes, such as SOD and GSH-Px, which reduces the accumulation of ROS, thus mitigating oxidative stress-induced activation of HSCs. Future research should explore its potential applications in clinical liver disease treatment.

For reversing fibrosis, key coordinated mechanisms include modulating the inflammatory environment and reducing oxidative stress balance. Notably, GSH production is regulated by Nrf2, a potential target for managing hepatic fibrosis. As GSH influences numerous redox-sensitive transcription factors, which regulate inducible nitric oxide synthase (iNOS) expression, reduced GSH levels trigger iNOS expression in liver tissue. Research involving iNOS knockout mice and specific iNOS inhibitors examined the role of iNOS in liver fibrosis, revealing reduced liver fibrosis [26]. TGF-β1-mediated ROS increase inhibits Nrf2 signaling pathway, suggesting the role of the Keap1-Nrf2 system in the inhibition of TGF-β1 signaling pathway [27]. The insulin-like growth factor-1 (IGF-1), Keap1, and Nrf2 interrelation can be interpreted through the regulation of the TGF-β1/smad2/3 pathway to diminish ROS/inflammation and the capabilities of the associated molecules. Al-Harbi *et al.* reported that by stabilizing serum liver enzyme levels, attenuating oxidative stress markers, and inhibiting the release of tumor necrosis factor-α, they successfully mitigated CCl_4_-induced liver injury in rats [28]. Combined or singular SCFA usage can ameliorate the detrimental effects of thioacetamide (TAA), such as elevated liver enzyme levels and damaged SOD and CAT [29]. *AKK* treatment effectively enhances propionic acid metabolism, thus improving oxidative stress accumulation in HSCs, further affirming the therapeutic effect of *AKK* on these cells.

In summary, this study suggests that *AKK* is a potential therapeutic agent for liver fibrosis, acting by increasing propionic acid levels transported from the intestine to the liver. Treatment with propionic acid largely replicates the beneficial metabolic effects observed with *AKK* in mice, including inhibiting oxidative stress, improving the TGF-β1/SMAD2/3 axis, and enhancing propionic acid metabolism along the intestinal–liver axis (Graphic Abstract). Consequently, it ameliorates fibrosis caused by collagen deposition in intestinal and hepatic tissues. Our findings indicate that *AKK* alleviates liver fibrosis in mice by modulating propionic acid metabolism to improve intestinal–liver interactions.

### Limitations of the study

This study was limited by the use of a single animal model (CCl_4_-induced hepatic fibrosis mouse model) and a relatively short experimental duration. Further studies are needed to evaluate the long-term therapeutic potential and clinical translation of *AKK* and propionic acid. Additionally, mechanistic insights into the interaction between host immunity and microbial metabolites remain to be explored in greater detail.

## Materials and methods

### Materials

The study utilized various reagents and kits, including sodium propionate (MedChemExpress, HY-B1773A), 4’,6-diamidino-2-phenylindole (DAPI; Thermo, D3571), MDA, CAT, SOD, and GSH-Px assay kits (Thermo, EEA015; Jianglai Biotechnology, JL18163, JL12237, JL-T0879, respectively), propionic acid ELISA kit (Zhuocai Biotechnology, ZC-56375-J), and antibodies such as Col1α1 (Abcam, RRID: AB_138492), α-SMA (Abcam, RRID: AB_5694), TGF-β1 (Proteintech, 21898-1-AP), SMAD2, p-SMAD2, SMAD3, p-SMAD3 (Cell Signaling Technology, 5339, 18338, 9523, 9520, respectively), CAT (Atlas Antibodies, HPA051282), SOD (Fitzgerald, 70R-13887), Nrf-2 (Cell Signaling Technology, 80593-1-RR), Keap1 (Proteintech, 80744-1-RR), GPR41 (Proteintech, 66811-1-Ig), GPR43 (Proteintech, 19952-1-AP), and Trizol reagent (Beyotime, R0016).

### AKK

The type strain *AKK* (ATCC BAA-835^™^) was cultured under strict anaerobic conditions (85% N_2_, 10% H_2_, and 5% CO_2_) in a modified brain heart infusion (BHI) broth. The basal BHI medium (45 g/L; HuanKai Microbial Technology, Cat# HK-BHI045, Lot: 024053) was supplemented with gastric mucin (4 g/L; Yuanye Bio-Technology, Cat# S12065) and L-cysteine hydrochloride (10 mg/L; MedChemExpress, Cat# HY-Y0337). Cultures were incubated statically at 37°C within an anaerobic chamber (Coy Laboratory Products). Bacterial growth was monitored by measuring the optical density at 600 nm (OD_600_), and growth kinetics were characterized by plotting the growth curve. Bacterial cells were harvested during the mid-logarithmic growth phase (OD_600_ ≈ 0.6−0.8), as confirmed by viable plate counts on mucin-supplemented BHI agar. Subsequently, cultures were centrifuged at 4,000 *g* for 5 min at 4°C (Eppendorf 5810R). The pelleted cells were washed twice with phosphate-buffered saline (PBS) and resuspended in PBS to a final concentration of 1.0 × 10^8^ colony-forming unit (CFU) per milliliter. Cell suspensions were stored at 4°C for short-term use (< 24 h). All procedures, including inoculation, harvesting, and processing, were performed within the anaerobic chamber to maintain anoxic conditions.

### Animal experiments

Male C57BL/6 mice, aged 7–8 weeks and weighing 18–20 g, were accommodated in a specific pathogen-free environment at the Experimental Animal Center of Nantong University.

To establish the liver fibrosis model, mice were intraperitoneally injected with a CCl_4_/olive oil mixture (25% v/v CCl_4_) at a dosage of 5 μL/g body weight, administered twice weekly for 8 consecutive weeks. Following fibrosis induction, mice were randomly allocated into two treatment groups using a computer-generated randomi­zation protocol: vehicle group: received PBS by oral gavage; *AKK* treatment group: administered live *AKK* suspended in anaerobic PBS (1 × 10^8^ CFU/mL). Both groups underwent continuous treatment for 8 weeks. Bacterial suspensions (200 μL) or vehicle control were delivered via oral gavage every 48 h. All interventions were performed under aseptic conditions with strict maintenance of anae­robiosis for *AKK* preparations.

To induce the ABX model, mice with hepatic fibrosis were orally administered an ABX mixture consisting of ampicillin (0.2 g/L, MedChemExpress, CAS# HY-B0522), vancomycin (0.2 g/L, MedChemExpress, CAS# HY-B0671), neomycin sulfate (0.2 g/L, MedChemExpress, CAS# HY-B0470), and metronidazole (0.2 g/L, MedChemExpress, CAS# HY-B0318) for 1 week before the 8th week. This treatment was followed by continued administration of ABXs or *AKK* for a total of 8 weeks. The ABXs were dissolved in the drinking water. Additionally, to assess the therapeutic effect of propionic acid on mice with liver fibrosis, mice were provided with drinking water containing sodium propionate (400 mmol/L), or sodium chloride as the control.

### Plasma biochemistry analysis

Blood samples were collected in 1 mmol/L EDTA tubes to assess various biochemical markers, including liver-specific enzymes, including ALP, γ-GT, AST, and ALT; antioxidant enzymes, including SOD, CAT, and GSH-Px, as well as MDA and propionic acid.

### Histological examination

The tissues of interest were weighed and then dehydrated using a series of graded ethanol concentrations (70%−100%) before embedding in paraffin. Paraffin-embedded samples were sectioned at a thickness of 6 μm using a rotary microtome. Following collection, sections underwent staining with H&E, Masson’s trichrome, or Sirius Red for collagen deposition visualization in liver fibrosis, employing IHC targeting Col1α1. To assess intestinal barrier integrity, periodic acid-Schiff (PAS) staining was applied, along with evaluation of ZO-1 protein expression levels.

### qRT‑PCR

Total RNA was extracted using Trizol reagent following standard protocols. Subsequently, reverse transcription and cDNA synthesis were performed using the Vazyme Reverse Transcription Kit. SYBR Green (Vazyme) was used for cDNA amplification and quantification, following established procedures. Gene expression was normalized to *β-actin* levels. DNA was extracted from fecal samples using a kit (Solarbio), and collected DNA was processed according to the kit instructions for qRT-PCR, normalized against 16 s rRNA. All gene expression levels were calculated and quantified using the 2^−ΔΔCT^ method and the specific primer details can be found in Supplementary Table S1.

### Immunoblotting and immunofluorescence

Proteins were extracted from lysed cells or tissues, quantified, and then electrophoretically separated using SDS–PAGE before transfer onto a polyvinylidene fluoride (PVDF) membrane. To minimize nonspecific interactions, the membrane was blocked using 5% bovine serum albumin in Tris-buffered saline with Tween-20 for 30 min at ambient temperature, followed by incubation with the primary antibody overnight and the secondary antibody thereafter. Protein bands were detected using an ECL kit.

In immunostaining procedures, cells underwent fixation and permeabilization before overnight incubation with the primary antibody at 4°C. After primary antibody incubation, cells were washed and then co-stained with fluorescent secondary antibodies and 2 μg/mL DAPI for nuclear visualization. This preparation was incubated at 37°C for 1 h before imaging with a Thunder microscope (Leica, Germany).

### FITC-dextran tracer assay

Before the assay, mice were subjected to a 4-h period of fasting and dehydration. Following this, mice received an oral dose of FITC-dextran (Beyotime, ST2940) at 400  mg/kg. Four hours after administration, mice were euthanized for blood collection, and serum was subsequently extracted. The serum fluorescence was measured using a Spark multimode microplate reader (Tecan, USA) at an excitation wavelength of 488 nm and emission wavelength of 525 nm. A standard curve was constructed to determine the concentration of FITC-dextran in the samples.

### 16S rRNA analysis

Total DNA was extracted from fecal samples, and the V3–V4 regions of the 16S rRNA gene were amplified with a universal bacterial primer set, followed by sequencing on the Illumina MiSeq PE250 platform using paired-end sequencing (2 × 300 bp). Raw sequencing data were processed for merging, demultiplexing, and quality filtering through QIIME 2.0. OTUs were delineated at 97% similarity, utilizing the Greengenes database v13.5. To assess microbial composition variations among groups, PCA and principal coordinates analysis (PCoA) were performed based on Bray–Curtis distance using R software. Differential OTUs linked to various interventions were pinpointed using LEfSe analysis, with significance established by *P*-values < 0.05 and linear discriminant analysis (LDA) scores of at least 4.0.

### Metagenomic sequencing

DNA samples for metagenomic sequencing were meticulously selected based on a concentration exceeding 20 ng/μL and an optical density ratio of 1.8–2.0 at 260/280 nm wavelengths. These samples underwent sequencing library construction using the NEBNext^®^ Ultra^™^ DNA Library Prep Kit for Illumina by New England Biolabs, USA. Index codes were incorporated to track sequences back to their respective samples. The libraries were then sequenced on an Illumina NovaSeq platform, facilitated by Novogene Technology Co., Ltd, China, employing paired-end reads. Reads were realigned to these contigs using BWA-MEM, and the contig depths were quantified using Samtools along with the jgi_summarize_bam_contig_depths function in MetaBAT2. To explore differential gene expression, Kruskal–Wallis tests were utilized, with results visually represented in box plots. PCA was performed to analyze species abundance at the phylum level. Species that differed significantly in abundance across groups were identified through rank sum tests and further analyzed using LDA to calculate LDA scores. Lastly, gene functional annotations were carried out using the DIAMOND Unigenes database applying a blastp evalue of ≤1e^− 5^, focusing on sequences with scores above 60 bits.

### Untargeted metabolomics and analysis

Metabolomic profiling of liver samples was conducted through liquid chromatography-tandem mass spectrometry (LC-MS/MS) by Wekemo Technology Group Co., Ltd, Shenzhen, China. The procedure initiated by pulverizing 100 mg liver tissue with liquid nitrogen, followed by the addition of 500 μL of chilled 80% methanol and vortex mixing. The samples were then incubated on ice for 5 min before being centrifuged at 15,000 *g* for 20 min at 4°C, with the supernatants subsequently filtered through a 0.22-μm membrane in preparation for LC-MS/MS analysis.

The LC-MS/MS analysis utilized a Thermo Fisher Scientific Vanquish UPLC system and an Orbitrap Q ExactiveTMHF-X mass spectrometer, employing a Hypesil Gold column (100 mm × 2.1 mm, 1.9 μm). The injection flow rate was maintained at 0.2 mL/min over a 12-min linear gradient.

Data processing was conducted using Compound Discoverer 3.1 (CD3.1, Thermo Fisher), involving peak alignment, detection, and quantification of metabolites. Peak intensities were normalized to the total spectral intensity, and subsequently matched against mzVault, MassList, and mzCloud databases for compound identification.

### Cell experiments

Human immortalized HSCs (LX2 cell line) were cultured in complete Dulbecco’s modified Eagle’s medium (DMEM) supplemented with 10% fetal bovine serum and 1% penicillin–streptomycin (Beyotime, Shanghai, China). Cells were maintained at 37°C in a humidified atmosphere with 5% CO_2_. Upon reaching 80% density, a 12-h serum starvation was conducted, followed by treatment with 10 ng/mL TGF-β1. To examine the activation and antioxidant stress response of LX2 cells exposed to TGF-β1 and sodium propionate, different concentrations of sodium propionate (2.0, 4.0, and 8.0 nmol/L) were used to treat LX2 cells for 24 h. Subsequently, cells were collected and lysed, and supernatants were obtained. Levels of MDA, SOD, and CAT were determined using commercially available assay kits.

### RNA interference *in vitro*

The infection protocol for lentiviral interference vectors targeting *GPR41* and *GPR43* was performed in accordance with the guidelines provided by Shanghai Sangon Biotech. LX2 cells were seeded at a density of 2 × 10^5^ cells/well in a six-well plate and cultured overnight. Subsequently, the cells were infected with lentiviral vectors at a multiplicity of infection (MOI) of 5 and incubated for 24 h. GFP-positive cells was assessed using fluorescence microscopy. The shRNA lentiviral vectors were procured from Sangon Biotech.

### Statistical analysis

Data in this study were reported as mean ± SEM. Experimental outcomes were determined using a two-tailed unpaired Student’s *t*-test or one-way ANOVA. For the analysis of fecal microbiota at the family and genus levels, the Wilcoxon rank-sum test was applied. Specific statistical methods are detailed in the figure legends. All statistical analyses were conducted using GraphPad Prism V.8.23. A *P*-value < 0.05 was deemed statistically significant, whereas a *P*-value > 0.05 was not considered significant.

**Supplementary data ** 

Supplementary data is available at *Life Metabolism* online.

## Supplementary Material

loaf036_Supplementary_Data

## Data Availability

All the data supporting the findings of this study are available within the supplementary material and corresponding authors.
