# Supplementary material for: Propionic acid secreted by Akkermansia muciniphila alleviates hepatic fibrosis by antioxidant regulation across the gut–liver axis
Source: Life Metab. 2025 Oct 8;5(1):loaf036. doi: 10.1093/lifemeta/loaf036 (PMC12857572; doi:10.1093/lifemeta/loaf036)
Supplement: loaf036_Supplementary_Data [file loaf036_supplementary_data.docx]

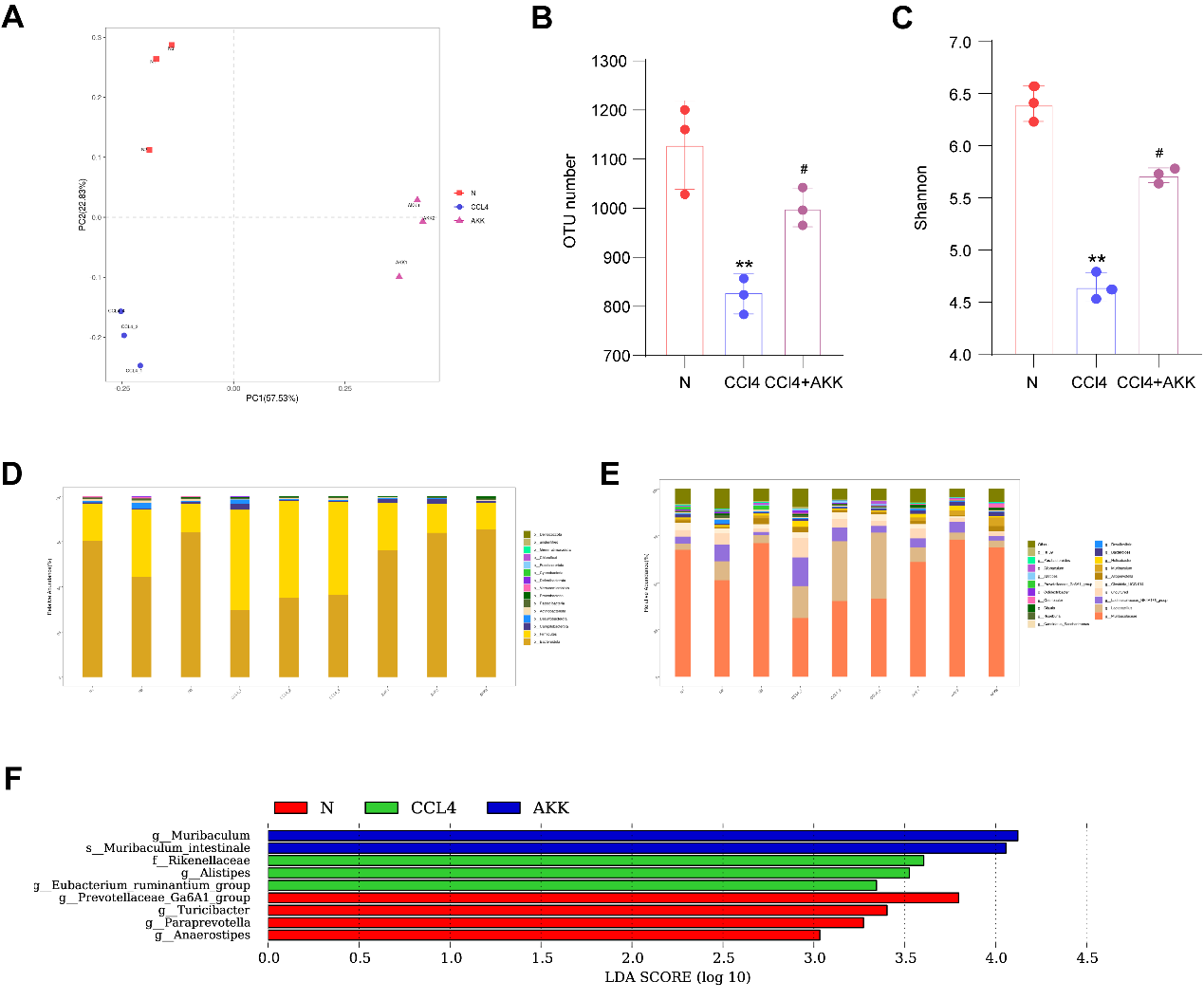


**Supplementary Figure S1** Effect of *AKK* on gut microbiota composition in mice with hepatic fibrosis. (a) PCoA plots of gut microbiota (*n* = 3). (b and c) Gut microbiota species (indicated by operational taxonomic units (OTUs)) (b) and diversity determination (indicated by the Shannon index) (c). (d and e) Relative abundance of intestinal microbiota at the genus level (d) and phylum level (e) (*n* = 3). (f) LEfSe analysis and LDA score based on LEfSe analysis (LDA > 2) (*n* = 3). Data are presented as the mean ± SEM. Statistical significance was determined using ANOVA for multiple-group comparisons. ^*^*p* < 0.05, ^**^*p* < 0.01, ^***^*p* < 0.001, compared with the control group. ^#^*p* < 0.05, ^##^*p* < 0.01, compared with the model group.


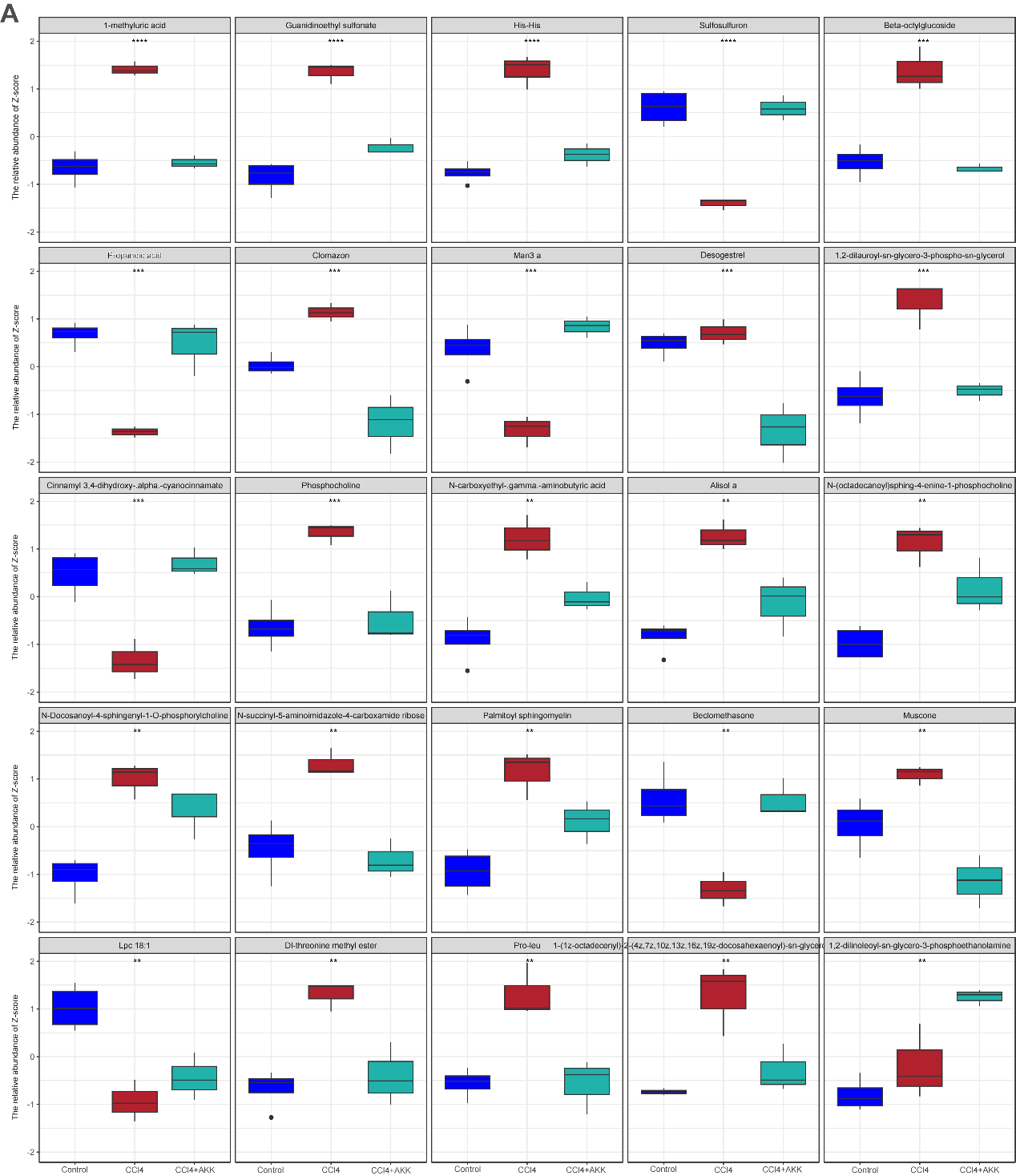


**Supplementary Figure S2** *AKK* improves the metabolic composition of mice with liver fibrosis. Identification of significant differential metabolites in CCl_4_ or *AKK*-treated hepatic fibrosis mice was conducted by LC-MS analysis.


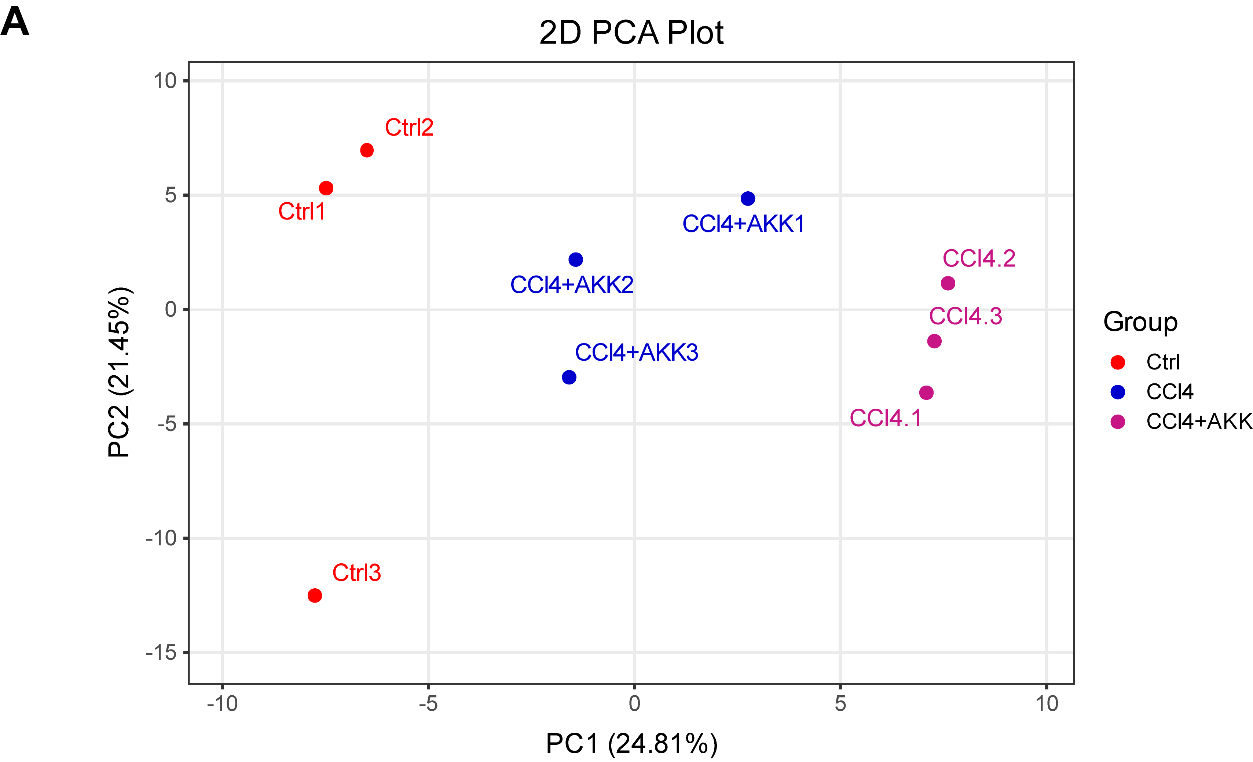


**Supplementary Figure S3** Supplementation with *AKK* enhances propionic acid-metabolizing microbiota and the expression of related enzymes. PCoA plots of gut microbiota are shown (*n* = 3)


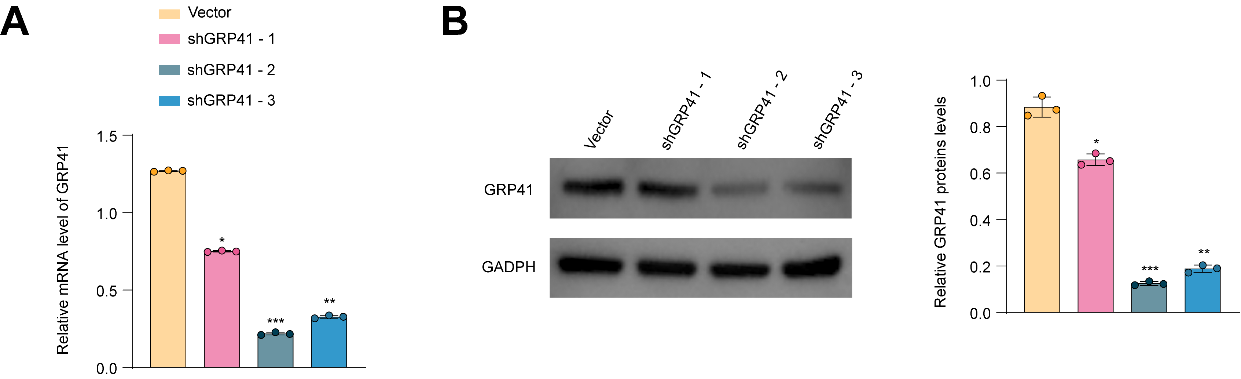


**Supplementary Figure S4** Validation of the knockdown efficiency of sh*GPR41* in LX2 cells. (a) RNA expression levels of *GPR41* quantified using qRT-PCR. (b) Protein levels of GPR41 evaluated using western blot analysis.


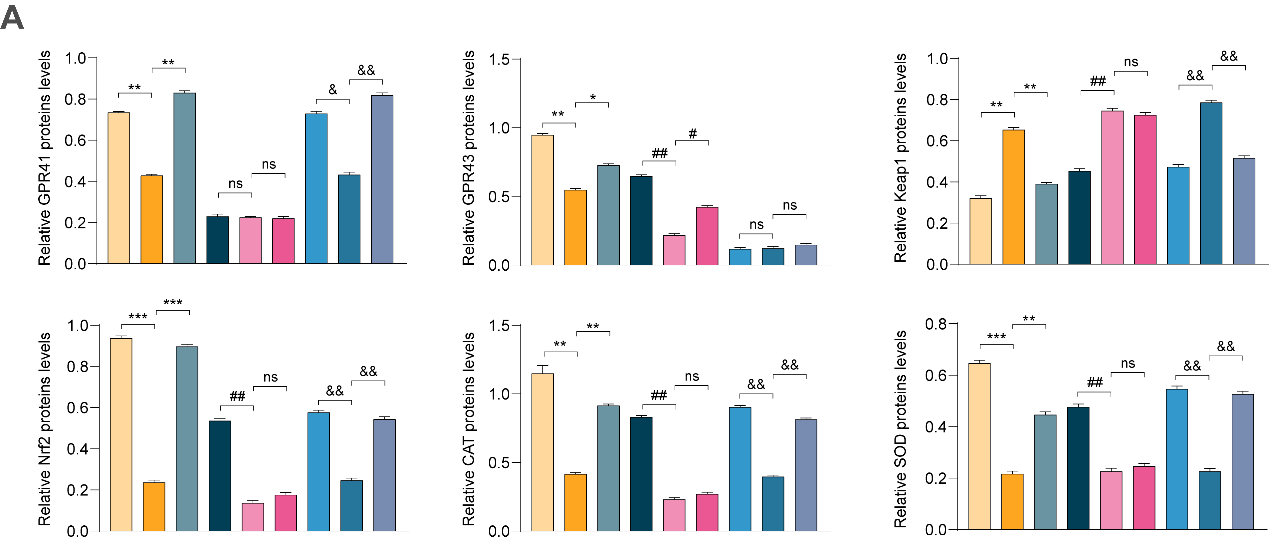


**Supplementary Figure S5** Protein expression quantification and evaluation of the effects of TGF-β, GPR41 inhibitor (SHB), GPR43 inhibitor (GLPG0974), and propionic acid pretreatment on oxidative stress-related Keap1/Nrf2 signaling pathway in LX2 cells using immunoblot analysis.

**Supplementary Table S1** Primer sequences for the amplification.

| Gene | Forward primer（5′ → 3′） | Reverse primer（5′ → 3′） |
| --- | --- | --- |
| *Collagen-I* | GCTCCTCTTAGGGGCCACT | CCACGTCTCACCATTGGGG |
| *TIMP-1* | GCAACTCGGACCTGGTCATAA | CGGCCCGTGATGAGAAACT |
| *Tgfb1* | GAGAAGCGGTACCTGAACCC | GAGAAGCGGTACCTGAACCC |
| *ZO-1* | ACCTCTGCAGCAATAAAGCAG | GAAATCGTGCTGATGTGCCA |
| *Claudin-1* | TGCACATGCCTTCAACTGTTCT | ACAAAACCTTGCTCTGCTGAAG |
| *SLC5A8* | GCTGCTGCTTCTGCTTCTTC | CAGCAGCAGCAGCAGTAACC |
| *SLC16A3* | TGACCTGGTGCTGGTTCTTA | TCCTTGTGACCAGCAGCTTT |
| *GRP41* | AGTCGCCTGGTGTGGATACTGAG | GCCGAAGCAGACGAAGAAGATGAG |
| *β-actin* | GGCTGTATTCCCCTCCATCG | CCAGTTGGTAACAATGCCATGT |
| *Akkermansia muciniphila* | CAGCACGTGAAGGTGGGGAC | CCTTGCGGTTGGCTTCAGAT |
| *16S* | ACTCCTACGGGAGGCAGCA3 | GGACTACHVGGGTWTCTAAT |
